# Pathology of Diabetes Insipidus

**Published:** 1897-09

**Authors:** 


### PATHOLOGY OF DIABETES INSIPIDUS.

The pathology of diabetes insipidus still remains obscure, and this is more especially the case where this condition of diabetes arises suddenly. Diabetes insipidus of sudden onset is not uncommon as a result of intracranial lesions frequently traumatic in origin. Dr. Hendrie Lloyd records a case of this disease arising in a young man of twenty-four, "after an apoplectiform or epileptiform attack, the exact nature of which remained obscure." The main interest of the case, however, turns on the fact that the lesion, whatever its nature, caused also paralysis of the sixth nerve on one side only, and that there was no evidence apparently of any other and more widespread lesion. The fact that no other palsy occurred, and that the seventh nerve was not affected, pointed to the conclusion that the damage to the fourth ventricle must have been limited to a very small area. The urine contained no sugar, and this is in accord with the experimental fact that the puncture of the medulla may cause an increase in the flow of urine without causing glycosuria; in fact, the situation of the two punctures is somewhat different, so that the experimenter can produce polyuria alone, or polyuria and glycosuria at will. This case of Dr. Lloyd's is also interesting as serving to localize the so-called center controlling the flow of urine to the vicinity of the nucleus of the sixth nerve. These cases of diabetes insipidus arising suddenly are, however, not always dependent upon a severe cerebral lesion, such as apparently occurred in this case, although frequently seen in cases of hemorrhage, etc., in the neighborhood of the fourth ventricle. Thus the writer has seen a case of aortic disease where suddenly one day well-marked diabetes insipidus came on and persisted for many weeks—in fact as long as the patient was under observation; and in this case, although it is possible that embolism of some small area of the medulla occurred, yet there were no general or local symptoms to point to any cerebral lesion, and still less to one of the pons or medulla.—*Prac.*

